# Metal-Free One-Pot Multi-Functionalization of Unsaturated Compounds with Interelement Compounds by Radical Process

**DOI:** 10.3390/molecules28020787

**Published:** 2023-01-12

**Authors:** Yuki Yamamoto, Akiya Ogawa

**Affiliations:** Department of Applied Chemistry, Graduate School of Engineering, Osaka Prefecture University, Osaka 599-8531, Japan

**Keywords:** heteroatoms, interelement compounds, metal-free conditions, radical reaction, unsaturated compounds, functionalization

## Abstract

In recent years, the importance of “environmentally friendly manufacturing” has been increasing toward the establishment of a resource-recycling society. In organic synthesis, as well, it is becoming increasingly important to develop new synthetic strategies with resource conservation and the recycling of elemental resources in mind, rather than just only synthesis. Many studies on the construction of frameworks of functional molecules using ionic reactions and transition-metal-catalyzed reactions have been reported, but most of them have focused on the formation of carbon–carbon bonds. However, it is essential to introduce appropriate functional groups at appropriate positions in molecules in order for the molecules to express their functions, and furthermore, the highly selective preparation of multiple functional groups is considered important for the creation of new functional molecules. In this review, we focus on radical reactions with high functional group selectivity and overview the recent progress in practical methods for the simultaneous introduction of multiple functional groups and propose future synthetic strategies that emphasize the recycling of elemental resources and environmental friendliness.

## 1. Introduction

In recent years, “environmentally friendly manufacturing” has become increasingly important to construct a resource-recycling society. In particular, many efforts have been made to reduce wastes in the synthesis of functional molecules in the field of pharmaceuticals, biotechnology, and functional material chemistry, but there remain many challenges to be overcome. Many studies on the construction of the scaffolds of functional molecules using ionic reactions and transition-metal-catalyzed reactions have been reported, but most of them have focused on carbon–carbon bond formation [1,2,3,4,5]. However, in order for a molecule to express a special function, it is essential to introduce an appropriate functional group at an appropriate position in the molecule. For the creation of more innovative functional molecules, molecules with multiple carbon–heteroatom bonds are expected to be important candidates that can impart various functions. From this perspective, attention is focused on the development of innovative synthetic methods that simultaneously introduce multiple functional groups while ensuring the resource conservation of elemental resources [6,7,8,9].

The introduction of different heteroatoms into building blocks has been studied primarily on the basis of ionic reactions. The “substitution reaction”, which is often seen in ionic reactions, generates large amounts of wastes as byproducts. Recently, methods using transition metal catalysts have also been reported, but these methods involve significant costs for waste disposal and the removal of metal residue in the products. Therefore, the development of more practical and atom-efficient methods is desired [10,11,12].

**Scheme 1:**
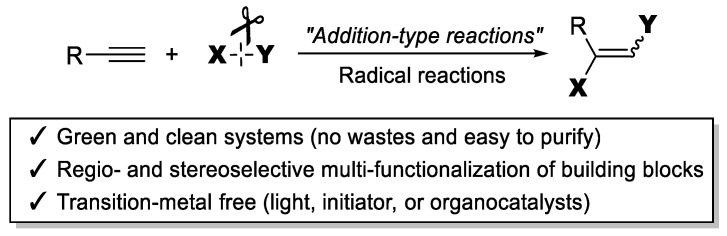
General concept for the utilization of heteroatom-centered interelement compounds in the radical multi-functionalization of building blocks.

Hence, this review focuses on metal-free reactions, especially radical reactions with excellent functional group selectivity. Among the radical reactions, “radical addition reactions” can incorporate all starting substrates into the product and can be regarded as a more atomically efficient synthetic method compared to substitution-type reactions involving the byproduction of leaving groups. Since each heteroatom has individual properties in radical reactions, highly selective multi-functionalization is possible by appropriately combining heteroatom reagents [13,14]. Interelement compounds, which have heteroatom–heteroatom linkages, are expected to be very effective for the simultaneous introduction of multiple heteroatom functional groups, because heteroatom-centered radicals can be generated by homolysis under photoirradiation [15,16]. Thus, molecular design using interelement compounds is highly expected to become an important fundamental technology for organic synthesis in the future (Scheme 1).

In this review, we present recent progress in the environmentally harmonized synthesis of carbon–heteroatom bonds by radical addition reactions to unsaturated compounds using group 13, 15, and 16 interelement compounds. Owing to their relatively strong dissociation energies, bond cleavage in the radical reactions of group 14 interelement compounds is disadvantageous. Instead, transition-metal-catalyzed addition reactions have been used effectively [17,18,19,20]. In the case of interelement compounds containing halogen, addition reactions usually proceeded by ionic pathways in preference to radical pathways. Therefore, group 14 and 17 interelement compounds are not dealt with here. This review also deals with future perspectives for radical reactions of interelement compounds.

## 2. The Group 16 Element (O, S, Se, and Te)-Centered Interelement Compounds in Radical Reactions

**Scheme 2:**
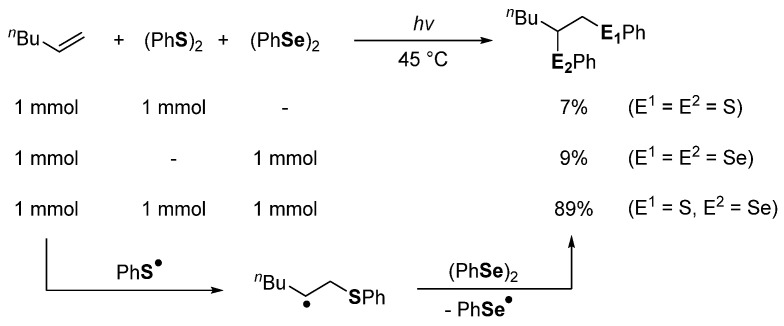
Photoinduced thioselenation of alkenes using (Ph**S**)_2_/(Ph**Se**)_2_−mixed system.

Previously, we developed a series of photoinduced addition reactions of organochalcogen interelement compounds to various unsaturated compounds [21,22,23,24,25,26,27]. During these studies, we noticed the combination of two kinds of interelement compounds is very important to induce efficient radical addition reactions. For example, diphenyl disulfide or diselenide alone could not add to alkenes efficiently; however, the use of them together successfully induced an efficient and regioselective thioselenation of alkenes (Scheme 2). 

This is because of the relatively higher reactivity of PhS• (*k*_PhS•_/*k*_PhSe•_ = ca. 10~50) and the relatively higher carbon-radical-trapping ability of (PhSe)_2_ (*k*_(PhSe)2_/*k*_(PhS)2_ = ca. 160). Therefore, PhS• selectively attacks the terminal position of alkenes, and the resulting *β*-phenylthio-substituted alkyl radicals are selectively captured by (PhSe)_2_ [28]. 

**Scheme 3:**
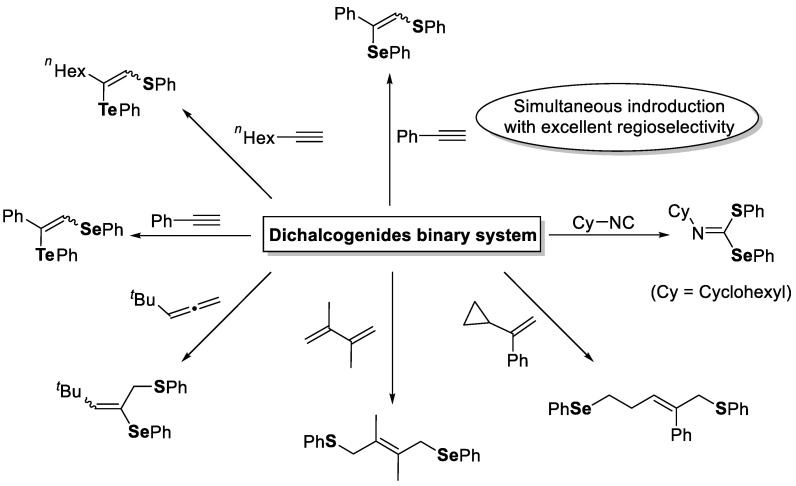
Dichalcogenide binary systems for multi-functionalization of unsaturated compounds under light.

As described here, the construction of mixed systems of organochalcogen interelement compounds effectively makes it possible to attain the simultaneous introduction of two chalcogen groups into unsaturated bonds with excellent regioselectivity. This concept can be applied to the radical addition reactions to a variety of unsaturated compounds, such as alkynes, allenes, conjugate dienes, vinylcyclopropanes, and isocyanides, as illustrated in Scheme 3 [29,30,31,32,33]. 

**Scheme 4:**
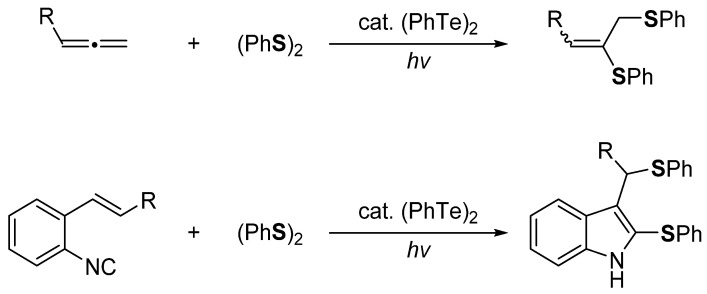
(PhTe)_2_-catalyzed dithiolation of allenes and *o*-ethenylaryl isocyanides.

Furthermore, a mixed system of (PhS)_2_ and (PhTe)_2_ is also effective to introduce two chalcogeno groups into unsaturated bonds; especially, the catalytic use of (PhTe)_2_ attained a novel dithiolation of allenes and *o*-ethenylaryl isocyanides (Scheme 4) [30,34].

**Scheme 5:**
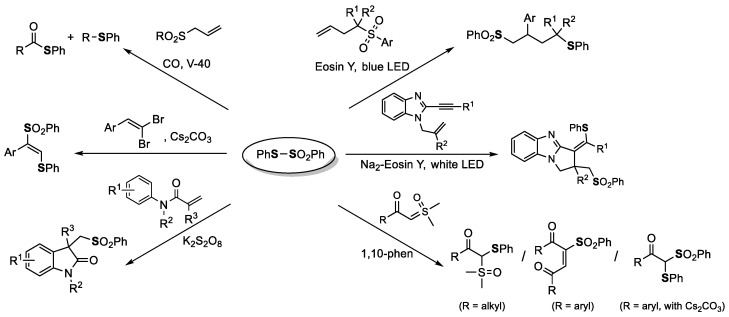
Some examples of radical cascade reactions using thiosulfonates (PhS–SO_2_Ph).

Thio- and selenosulfonate (PhS–SO_2_Ph and PhSe–SO_2_Ph) are reported to capture carbon radicals, and excellent examples using PhS–SO_2_Ph for the introduction of sulfur-centered functional groups into several building blocks are shown in Scheme 5. In the presence of radical initiators or under light, the corresponding sulfur-centered radicals are generated from PhS–SO_2_Ph, and sequential radical cascade reactions driven by trapping carbon radicals with them provide pharmaceutical and functional molecule scaffolds in one pot [35,36,37,38,39,40]. 

In sharp contrast, the combination of organic dichalcogenides ((RE)_2_, E = S, Se, Te) and molecular halogen (X_2_, X = Cl, Br, I) affords RE–X or RE–X_3_, which are well-known to work as electrophilic reagents. Therefore, it is difficult to induce radical reactions in preference to ionic reactions. However, the binary system of Rf–I and (PhSe)_2_ successfully attains the regioselective selenoperfluoroalkylation of alkynes by a radical pathway (Equation (1)) [41].
(1)
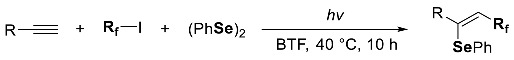


## 3. Phosphorus-Centered Interelement Compounds in Radical Reactions

Among the interelement compounds bearing a P–P single bond, diphosphine (R_2_P–PR_2_), diphosphine monoxides (R_2_P–P(O)R_2_), diphosphine monosulfides (R_2_P–P(S)R_2_), and diphosphine disulfides (R_2_P(S)–P(S)R_2_) can be used for the simultaneous introduction of two phosphorus functional groups into unsaturated compounds [42,43]. In particular, diphosphines bearing P^III^–P^III^ linkages have attracted many researchers to synthesize various bisphosphine ligands for organometallic chemistry and transition-metal-catalyzed reactions. For the highly efficient synthesis of phosphorus ligands, the simultaneous and stereoselective introduction of two phosphine units into unsaturated compounds is required, and effective methods by the addition of diphosphines have been developed over the past few decades [44]. 

Regarding the simultaneous introduction of two phosphine units by Ph_2_P–PPh_2_ via the ionic pathway, in 2006, Pringle et al. reported the stereospecific bisphosphination of activated acetylenes. In this reaction, a nucleophilic attack by Ph_2_P–PPh_2_ proceeds on an activated alkyne, such as dimethylacetylene dicarboxylate, and the subsequent intramolecular rearrangement of the generated zwitterionic intermediate gives the corresponding *vic*-diphosphinyl adduct as the exclusive *cis* isomer (Equation (2)) [45].
(2)
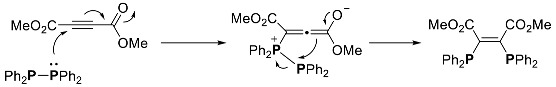


**Scheme 6:**
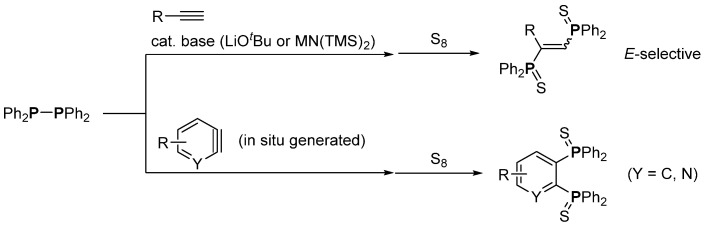
Simultaneous introduction of two phosphine units into unsaturated compounds with Ph_2_P–PPh_2_ via ionic reactions.

In 2017, Hirano and Miura et al. reported the stereoselective diphosphination of terminal alkynes by Ph_2_P-PPh_2_ in the presence of catalytic Bronsted bases [46]. In the case of terminal arylalkynes, the use of LiOtBu was found to be most effective, while the use of MN(TMS)_2_ (M = Li or Na) gave good results for aliphatic alkynes. This method gave the corresponding 1.2-diphosphinoethenes in good yields with excellent *E*-stereoselectivity. Moreover, they also developed the diphosphination of arynes, generated in situ from 2-(trimethylsilyl)aryl triflates and Bu_4_NPh_3_SiF_2_/or base, with Ph_2_P–PPh_2_ (Scheme 6) [47]. 

**Scheme 7:**
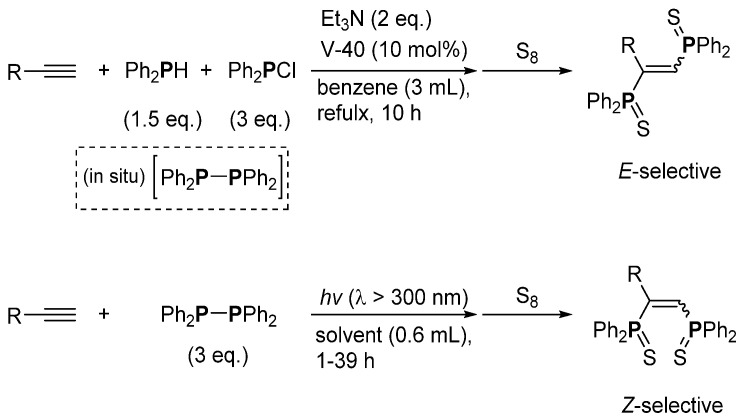
The radical addition of tetraphenyldiphosphine to alkynes.

Diphosphines are also important as precursors of phosphorus-centered radicals [48,49]. Diphosphines and their analogues can undergo P-P single bond homolysis upon photoirradiation or radical initiators to generate the corresponding trivalent and/or pentavalent phosphorus-centered radicals, such as R_2_P^•^ and/or R_2_P(O)^•^ [50,51,52]. An example of the radical addition of diphosphines to alkynes was reported by Yorimitsu and Oshima et al. in 2005 [53]. In this report, tetraphenyldiphosphine Ph_2_P–PPh_2_, generated in situ from diphenylphosphine and diphenylphosphine chloride in the presence of Et_3_N, was allowed to react with alkynes under reflux for 10 h in the presence of a radical initiator, resulting in the corresponding *vic*-1,2-bisphosphinoalkenes with excellent *E*-stereoselectivity. Around the same time, we also developed the radical addition reaction of Ph_2_P–PPh_2_ to alkynes under light. In this photoirradiation system, the corresponding *vic*-1,2-bisphosphinoalkenes were obtained with *Z*-selectively due to the photoinduced isomerization from *E*-adducts to *Z*-adducts (Scheme 7) [54]. 

**Scheme 8:**
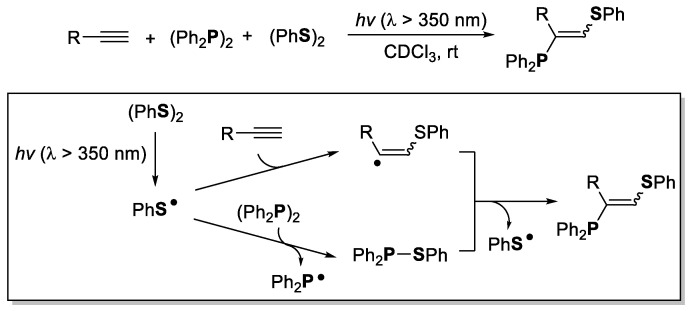
Photoinduced thiophosphination of alkynes using a (PhS)_2_/(Ph_2_P)_2_ binary system.

Furthermore, we also developed the photoinduced thiophosphination of alkynes by using the combination of (Ph_2_P)_2_ and (PhS)_2_ in 2008 (Scheme 8) [55]. The absorption based on the n-σ* transition in the UV-visible spectrum of (PhS)_2_ reached 375 nm, confirming that irradiation with the light of wavelengths over 350 nm leads to the homolytic cleavage of the S-S bond to form PhS^•^. The generated PhS^•^ reacted with alkynes and (Ph_2_P)_2_ to form vinyl radical species and Ph_2_P–SPh, respectively. Then, the generated vinylic radical was captured by Ph_2_P–SPh to afford the thiophophinated products with excellent regio- and stereoselectivity. Yorimitsu and Oshima et al. reported in 2008 that the reaction of Ph_2_P–SPh with alkynes could also be conducted by using radical initiators [56]. In 2009, they also developed the regio- and stereoselective synthesis of 1-aryl-1-thio-2-thiophosphinylethene derivatives using Ph_2_P(S)–SR analogues, with catalytic peroxide as the radical initiator [57].

**Scheme 9:**
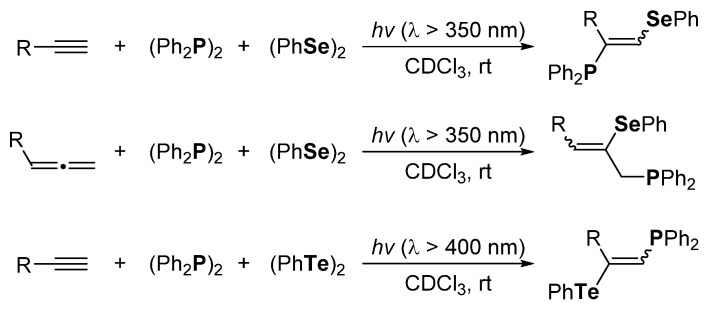
Photoinduced selenophosphination and phosphinotelluration.

We next focused on the photoinduced selenophosphination and phosphinotelluration of alkynes. The results are summarized in Scheme 9. The combination of (Ph_2_P)_2_ and (PhSe)_2_, or (PhTe)_2_ with alkynes, or allenes successfully provided the corresponding 1,2-adducts with excellent regio- and stereoselectivity via the formation of Ph_2_P–ChPh (Ch = Se, Te) under light. In the case of the phosphinotelluration of alkynes, the phosphorus group was introduced to the terminal position, and the PhTe-group was introduced to the internal position, in sharp contrast to thiophosphination and selenophosphination. These results clearly indicated that the relative reactivity toward unsaturated bonds could be estimated as follows: PhS• > PhSe• > Ph_2_P• > PhTe• [58,59]. 

**Scheme 10:**
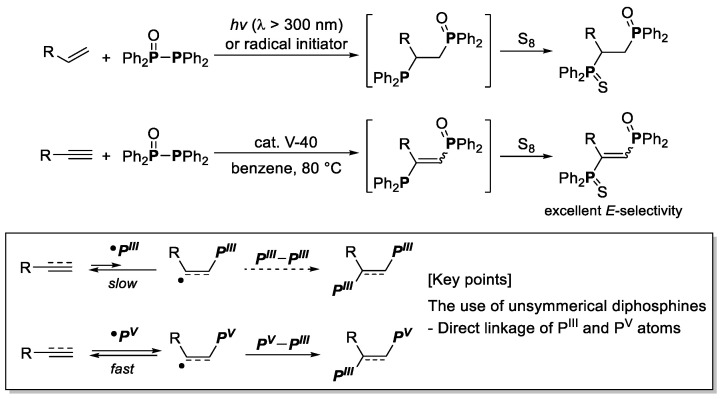
Radical phosphinylphosphination of alkenes and alkynes, using tetraphenyldiphosphine monoxide as the phosphorus interelement compounds.

Although the radical addition of (Ph_2_P)_2_ to alkynes and some application with the combination of dichalcogenides could be conducted, the addition reaction of (Ph_2_P)_2_ to alkenes hardly proceeded in the presence of radical initiators or under photoirradiation. This might be because of the instability of the *β*-heteroatom-substituted alkyl radical generated from alkene compared to that of the *β*-heteroatom-substituted alkenyl radical generated from alkyne. In Section 2, we pointed out that the use of unsymmetric interelement bonds is very effective for the radical addition to unsaturated bonds. Thus, we selected tetraphenyldiphosphine monoxide, Ph_2_P(O)–PPh_2_, as an unsymmetrical diphosphine. This diphosphine is composed of two types of phosphorus units, namely trivalent and pentavalent phosphorus groups. The higher reactivity of the pentavalent phosphorus radical compared to the trivalent one, and the higher carbon radical capturing ability of the trivalent phosphorus group compared to the pentavalent one, effectively caused the desired radical addition to alkenes [60,61]. This method could also be applied to the addition of Ph_2_P(O)–PPh_2_ to alkynes, which proceeded smoothly in the presence of catalytic V-40 as the radical initiator, affording the corresponding 1-diphenylphosphinyl-2-diphenylthiophosphinyl-1-alkenes in good yields with excellent regio- and stereoselectivity (*E*/*Z* = 95:5–99:1) (Scheme 10) [62].

**Scheme 11:**
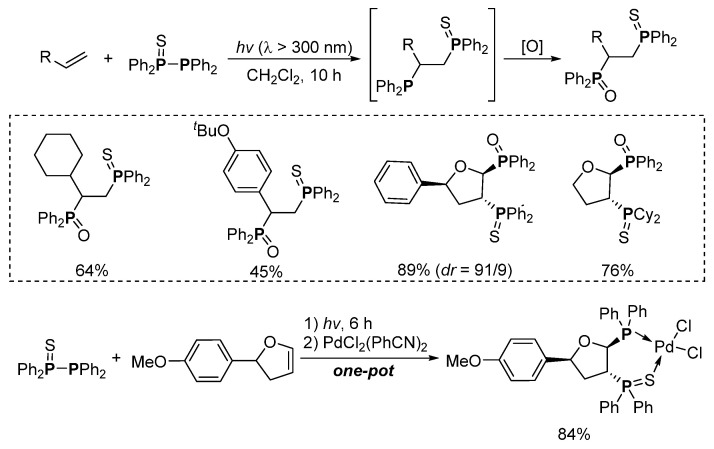
Photoinduced bisphosphination of alkenes, using tetraphenyldiphosphine monosulfide as the phosphorus interelement compounds.

In the case of Ph_2_P(O)–PPh_2_, however, the radical addition reaction required a long photoirradiation time (40 h) to obtain the adducts in adequate yields. This is because the cutoff of the absorption of Ph_2_P(O)–PPh_2_ reached only 318 nm. As the result, near-UV and visible light irradiation was less effective for the homolytic cleavage of the P–P bond of Ph_2_P(O)–PPh_2_. Thus, we selected tetraphenyldiphosphine monosulfide, Ph_2_P(S)–PPh_2_, the absorption cutoff of which reached 345 nm. The effective reactivity of Ph_2_P(S)–PPh_2_ was observed, and the corresponding *vic*-diphosphinyl adducts were obtained in good yields with a shorter photoirradiation time (10 h). This method could also be applied to the five-membered cyclic alkenes, and a one-pot reaction of the *vic*-diphosphinyl adducts (in situ-generated) with PdCl_2_(PhCN)_2_ provided the corresponding Pd^II^ bisphosphane monosulfide complex in good yields (Scheme 11) [63].

**Scheme 12:**
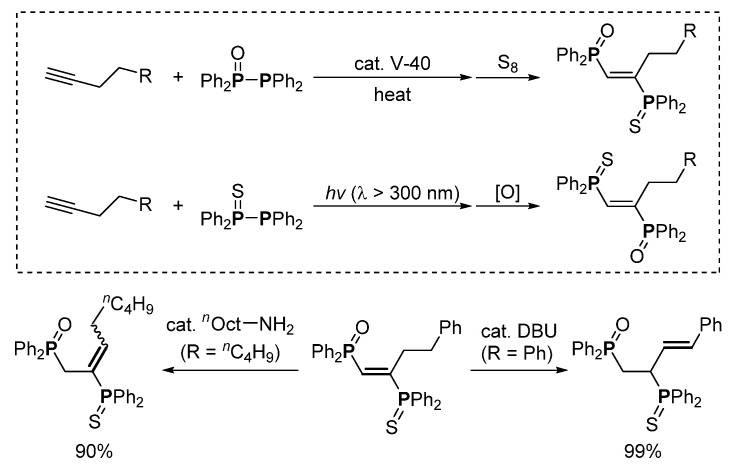
Regio-complementary synthesis of *vic*-1,2-bisphosphinoalkenes and its base-catalyzed double bond isomerization.

This system using Ph_2_P(S)–PPh_2_ successfully worked in the case of the photoinduced bisphosphination of alkynes. In this reaction, Ph_2_P(S)-units were introduced to terminal positions, and Ph_2_P(O)-units were located at the internal position, in contrast to the case of Ph_2_P(O)–PPh_2_. Hence, the regio-complementary introduction of Ph_2_P(S) and Ph_2_P(O) groups to alkynes could be attained, leading to a novel synthetic strategy for phosphine-centered functional molecules (Scheme 12). During our investigation of the further transformation of the *vic*-1,2-bisphosphinoalkenes obtained by the developed radical addition reaction of alkynes, we recently developed a novel base-catalyzed double-bond isomerization reaction of the bisphosphinated products. A catalytic amount of primary amines or DBU successfully catalyzed the isomerization, and the novel bisphosphinated vinyl compounds were obtained with completely regio- and stereoselectivities [64].

**Scheme 13:**
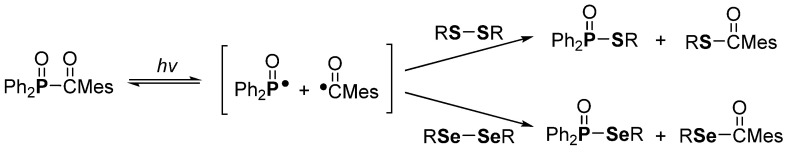
Photoinduced synthesis of P^V^(O)–chalcogen interelement compounds via radical generation from stable diphenyl(2,4,6-trimethylbenzoyl)phosphine oxide.

Diphosphines, which have trivalent phosphorus groups, are moisture- and air-sensitive; therefore, the strict Schlenk technique under an inert gas atmosphere is required. For the easy handling of phosphorus interelement compounds, it is expected to be effective to use interelement compounds that do not contain trivalent phosphorus groups, or, in other words, that are composed of pentavalent phosphorus groups. From this viewpoint, we developed the preparation of various kinds of stable phosphorus-centered interelement compounds, which have pentavalent phosphorus groups. For example, a photoinduced coupling reaction of diphenyl(2,4,6-trimethylbenzoyl)phosphine oxide (TMDPO) with (PhCh)_2_ (Ch = S, Se) was reported. Under photoirradiation, the homolytic cleavage of the P^V^(O)–C(O) bond in TMDPO occurred to form Ph_2_P(O)^•^ and MesC(O)^•^. Then, the formed radicals reacted with dichalcogenides via S_H_2 reaction or radical coupling pathways, and the corresponding thio- or selenophosphinates and thio- or selenoesters, respectively, were obtained in excellent yields (Scheme 13) [65]. 

**Scheme 14:**
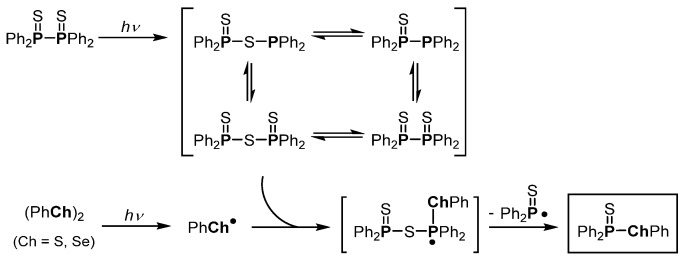
Direct construction of P^V^−Ch bonds via photoinduced radical reaction with tetraphenyldiphosphine disulfide and dichalcogenides.

Interestingly, when the photoinduced reactions of tetraphenyldiphosphine disulfide with an equivalent amount of diphenyl disulfide or diselenides, *S*- or *Se*-phenyl diphenylphosphinodithioates, respectively, were obtained in excellent yields as the sole products (Scheme 14). The mechanistic investigation revealed that the reaction proceeded via the photoinduced isomerization of tetraphenyldiphosphine disulfide, Ph_2_P(S)–P(S)Ph_2_, to generate an equilibrium mixture of Ph_2_P(S)–S–PPh_2_, Ph_2_P(S)–PPh_2_, Ph_2_P(S)–S–P(S)Ph_2_, and Ph_2_P(S)–P(S)Ph_2_. Among them, Ph_2_P(S)–S–PPh_2_ mainly acted as the carbon-radical-trapping reagent at the trivalent phosphorus site (Scheme 14). The P^V^–Ch bond formation usually proceeds via ionic pathways, using air- and moisture-sensitive reagents, and there has been no example of a radical method for the preparation of P^V^–Ch bonds. Therefore, the developed method will contribute to the further utilization of phosphorus-centered interelement compounds in organic synthesis [66].

**Scheme 15:**
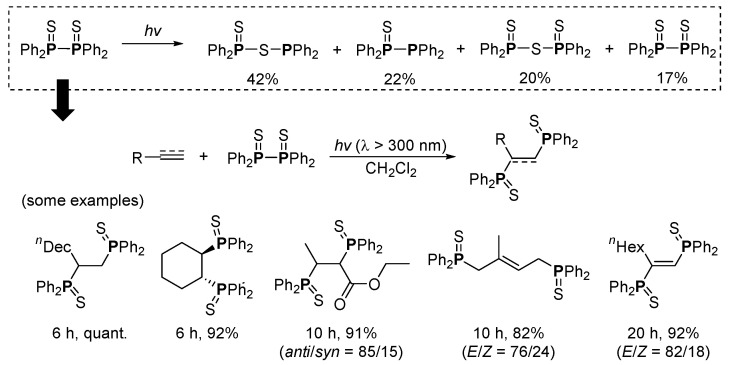
Photoinduced bisphosphination using tetraphenyldiphosphine disulfide.

Furthermore, the photoinduced bisphosphination of alkenes, alkynes, and conjugate dienes, using moisture- and air-stable Ph_2_P(S)–P(S)Ph_2_, was developed. Upon photoirradiation, the homolysis of a P^V^–P^V^ bond occurred to generate Ph_2_P(S)^•^, which added to the unsaturated bond. The resulting carbon radical was captured with Ph_2_P(S)–S–PPh_2_ at the trivalent phosphorus site, affording the corresponding *vic*-1,2-bisphosphinoalkanes in excellent yields in a short reaction time (4–10 h) (Scheme 15) [67].

## 4. Boron-Centered Interelement Compounds in Radical Addition Reactions 

Cross-coupling reactions using boron as a coupling partner are the most important carbon–carbon bond formation method in organic synthesis and are widely used for a wide range of molecular transformations to synthesize pharmaceuticals, functional materials, etc. Along with this, the development of reactions to introduce boron functional groups into organic molecules is becoming increasingly important. Recently, there has been a strong demand for the development of green synthetic methods with high atom efficiency and low environmental impact. From this perspective, addition reactions in which all starting substrates are ideally incorporated into the product are becoming more important than substitution reactions in which leaving groups are always byproducts. Diboron (B–B), haloboron (B-X), and silylboron (B-Si) are widely used as interelement compounds containing boron, and many excellent reactions using transition metal catalysts and bases have been reported [68,69,70,71,72,73,74]. The value of these reactions in organic synthesis is unassailable, but on the other hand, from the standpoint of environmental compatibility, the use of stoichiometric or higher amounts of bases leads to an environmental burden, and the use of metal catalysts requires the costly removal of trace amounts of metals in the products. Therefore, the development of the “metal-free” synthesis of boron compounds is expected to become more important in the future. In this section, we review the radical addition reactions and recently developed metal-free ionic reactions of boron-containing interelement compounds.

Diborons, the interelement compounds with B–B direct linkages, have been utilized as the boron sources in many reactions [75,76]. The first example of the metal-free diboration of unsaturated compounds with X_2_B–BX_2_ (X = F, Cl) was reported in 1959 [77]. After some related works [78,79], the metal-free diboration of alkenes, an allene [80], and conjugate enones [81,82,83] by the activation of diborons with Lewis bases was reported by Gulyás and Fernández. In 2014, Hirano and Uchiyama reported the *trans*-diborylation of propargylic alcohols in the presence of butyllithium [84]. Furthermore, Ohmiya and Sawamura reported the phosphine-catalyzed diboration or silaboration and LiO*^t^*Bu-catalyzed diboration of electron-deficient alkynes [85,86]. After these pioneering works, there were many examples of such base-catalyzed diboration of unsaturated compounds. In these reactions, alkali metal alkoxides [87,88,89], amines [90,91,92], and butyllithium [93,94,95,96] were used as bases. 

**Scheme 16:**
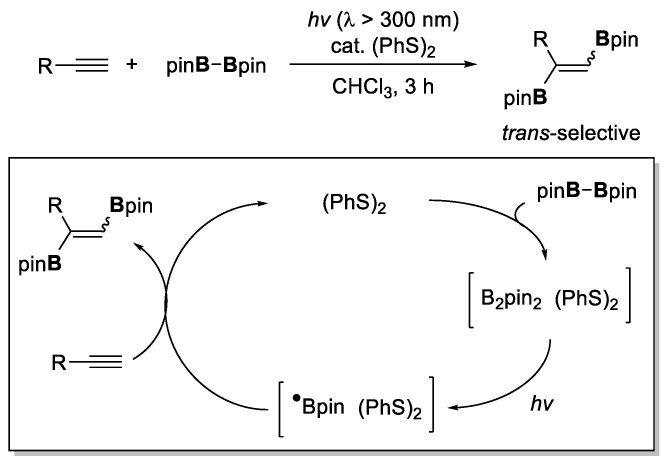
Organosulfide-catalyzed photoinduced diboration of alkynes.

**Scheme 17:**
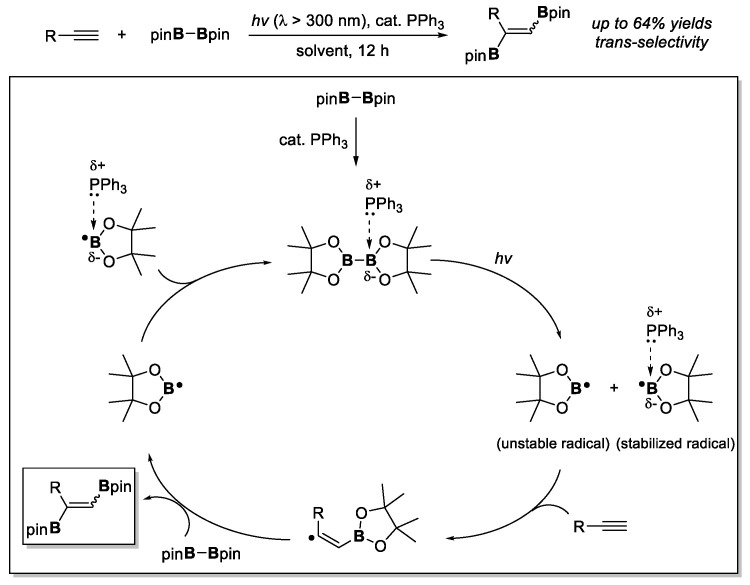
PPh_3_-catalyzed photoinduced metal-free diboration of alkynes.

On the other hand, we reported in 2015 that the radical diboration of alkynes successfully proceeded under light in the presence of catalytic (PhS)_2_, and the corresponding double borylation products were obtained in good yields with good *trans*-selectivity. The mechanistic investigation of the reaction by electron spin resonance (ESR) spectroscopy revealed that a novel boryl-centered radical was firstly generated (Scheme 16) [97]. Furthermore, the radical diboration of alkynes also proceeded in the presence of catalytic amount of PPh_3_ instead of (PhS)_2_, and the corresponding *vic*-diborated products were obtained in moderate yields with excellent *trans*-selectivity (Scheme 17) [98].

**Scheme 18:**
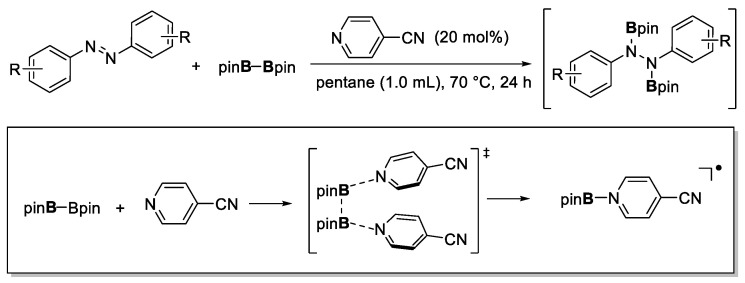
Homolytic cleavage of a B–B bond, using 4-cyanopyridine as the Lewis base.

Zhu and Li et al. reported the homolytic cleavage of boron–boron bonds by the cooperative catalysis of Lewis bases in 2016. In this system, the addition of a catalytic amount of 4-cyanopyridine was effective, and the authors suggested that the catalyst might act as a single-electron acceptor in the homolytic cleavage of boron–boron bonds. They also demonstrated the application of this system to the catalytic reduction of azo-compounds via the radical diboration of azoarenes, and the corresponding products were obtained in good to excellent yields (Scheme 18) [99,100,101].

**Scheme 19:**
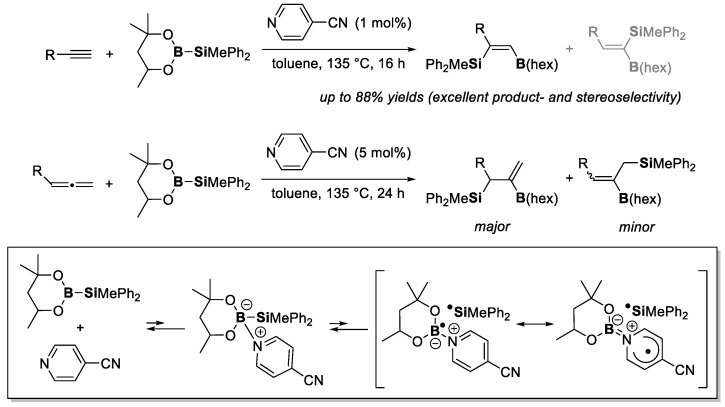
4-Cyanopyridine-catalyzed radical silaboration of alkynes and allenes.

The use of 4-cyanopyridine as the catalyst is also effective for the homolytic cleavage of boron-centered interelement compounds [102]. For example, Ohmura and Suginome et al. reported the 4-cyanopyridine-catalyzed radical silaboration of alkynes in 2019. The B–Si bond could be activated under heat, and the corresponding boryl radical was stabilized by the catalyst. The author suggested that the generated more nucleophilic pyridine–boryl radical preferentially attacked the more electron-deficient alkyne terminal position, and the formed vinyl radical was smoothly captured by the silyl radical to form the corresponding products with excellent product- and stereoselectivity. In addition, the system could be applied to the silaboration of allenes (Scheme 19) [103,104,105].

Another approach for the introduction of boron-centered functional groups into unsaturated compounds is the use of these boryl groups in combination with other reactive radical species [106]. In 2018, Studer et al. reported the metal-free 1,2-carboboration of inactivated alkenes, using the combination of perfluoroalkyl iodides and diborons as the carbon radical precursors and carbon-radical-trapping agents, respectively (Equation (3)). Under blue LED light irradiation, the carbon-centered radical, in situ-generated via C–I bond homolysis, first reacted with alkenes, and the formed secondary alkyl radical could be trapped with diborons to result in the corresponding adducts in good yields [107].
(3)
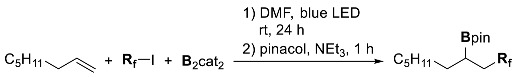


**Scheme 20:**
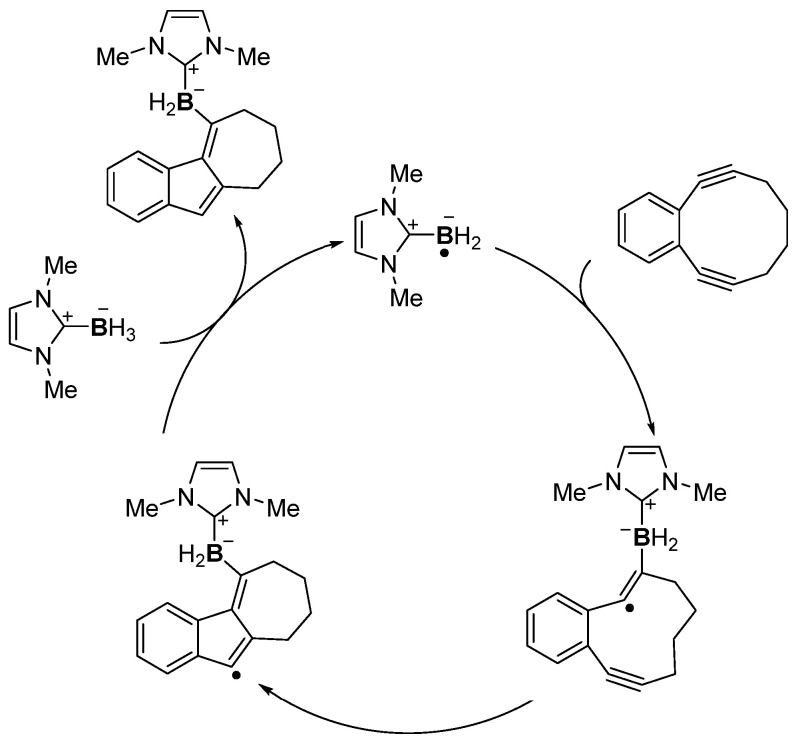
Borylative radical cyclization of strained cyclic diynes using NHC-boranes.

*N*-Heterocyclic carbene boranes (NHC-boranes) can be activated to form the corresponding NHC-boryl radicals, which can be used for radical cascade reactions [108,109,110,111,112]. A pioneering work of radical borylations using NHC-boranes and catalytic initiators was reported by Curran and Taniguchi et al. in 2017. In this reaction, benzo[3,4]cyclodec-3-ene-1,5-dienes, a strained cyclic diene, could be transformed to 5-borylated 6,7,8,9-tetrahydrobenzo[*a*]azulene via (1) the generation of NHC-boryl radicals from NHC-boranes by hydrogen abstraction using initiators, (2) the addition of the boryl radicals to form alkenyl radicals, (3) following transannular cyclization, and (4) a hydrogen atom transfer (HAT) reaction from the NHC-boranes (Scheme 20) [113]. The NHC-boranes/radical initiator conditions could be applied to the radical borylation/cyclization cascade of 1,6-enynes for the synthesis of boron-handled hetero- and carbocycles, which was reported by Wang et al. in 2017 [114].

For photoinduced radical borylation using NHC-boranes, the use of photocatalysts is also effective. In 2022, Wang et al. reported that visible light induced the regioselective hydroborylation of *α*,*β*-unsaturated carbonyl compounds with NHC-boranes, using (PhS)_2_ as the photocatalyst. This system could be conducted by blue LED light irradiation, and a variety of *α*-borylcarbonyl compounds could be obtained in good to excellent yields (Equation (4)) [115].
(4)
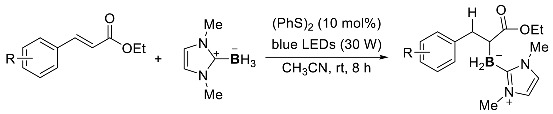


**Scheme 21:**
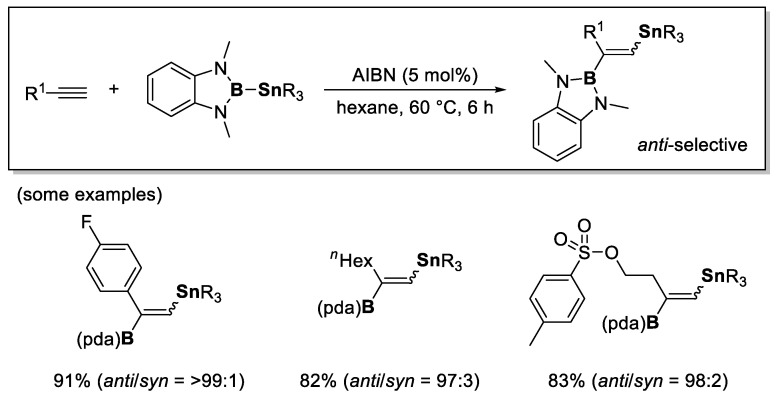
*anti*-Selective radical borylstannylation of alkynes based on the molecular design of borylstannanes.

Concerning the utilization of the interelement compounds with boron–Group 14 element linkages, Nishimoro and Yasuda et al. reported in 2022 that the *anti*-selective radical borylstannylation of alkynes successfully proceeded with (*o*-phenylenediaminato)borylstannanes. The authors estimated that the vacant *p*-orbital of the boron in (*o*-phenylenediaminato)borylstannane conjugated with the orbital of the phenylenediamine structure, and the efficient delocalization lowered the energy level of the LUMO; this allowed a large interaction with the SOMO of the alkyl radical (from initiators), and the radical borylation and stannylation took place smoothly. In addition, the S_H_2 reaction with generated vinyl radicals and (*o*-phenylenediaminato)borylstannane proceeded in a manner that avoided steric repulsion, and the corresponding products were obtained with *anti*-selectively (Scheme 21) [116,117].

## 5. Summary and Conclusions

This review focuses on the recent developments in the multi-functionalization of unsaturated compounds by radical addition reactions using interelement compounds. In recent years, radical addition reactions of interelement compounds have been energetically studied, and their environmentally benign and high atomic efficiency have attracted much attention as alternatives to transition-metal-catalyzed reactions and ionic reactions. In order to construct the important molecule scaffolds for pharmaceuticals and functional materials, the development of the precise introduction of heteroatom-centered groups has been actively pursued, focusing on the characteristic features of heteroatoms. 

For the introduction of heteroatoms, compounds with heteroatom–hydrogen or heteroatom–halogen bonds have been mainly used as heteroatom sources. However, the application of various interelement compounds to radical addition reactions is more limited. For group 15 and 16 heteroatoms, the presence of (a) lone pair(s) can allow the homolysis of the interelement bonds via n-σ* transition induced by photoirradiation. In addition, radical addition reactions are more likely to occur in group 16 heteroatoms than in other 13–15 groups because the number of substituents is relatively small. On the other hand, with group 15 phosphorus, the number of substituents increases, so steric hindrance affects addition, and radical addition reactions are somewhat more difficult to control, because trivalent phosphorus is unstable to air and moisture. On the other hand, group 13 boron has an unoccupied orbital, so homolysis results in a five-electron radical species, which is difficult to generate and control. Thus, when a Lewis base with a lone pair is coordinated to the unoccupied orbital of boron, the radical species generated is seven-valent and can be controlled. In group 14 heteroatoms, there are no lone pairs or unoccupied orbitals, and the number of bonds is much higher, so steric hindrance is a serious problem for inducing addition. Therefore, the development of synthetic applications of these unused interelement compounds by radical reactions is expected to provide new synthetic strategies for organic synthesis and further expand the potential of main group element organic chemistry.

We hope that the knowledge on the utilization of radical reactions of interelement compounds presented in this review will contribute to the further development of organic synthesis and green chemistry in the future. 

## Data Availability

Not applicable.
